# A Secure and Efficient White-Box Implementation of SM4

**DOI:** 10.3390/e27010001

**Published:** 2024-12-24

**Authors:** Xiaobo Hu, Yanyan Yu, Yinzi Tu, Jing Wang, Shi Chen, Yuqi Bao, Tengyuan Zhang, Yaowen Xing, Shihui Zheng

**Affiliations:** 1 Beijing Smart-Chip Microelectronics Technology Co., Ltd., Beijing 102299, China; huxiaobo@sgchip.sgcc.com.cn (X.H.); yuyanyan@sgchip.sgcc.com.cn (Y.Y.); tuyinzi@sgchip.sgcc.com.cn (Y.T.); wangjing@sgchip.sgcc.com.cn (J.W.); 2 School of Cyberspace Security, Beijing University of Posts and Telecommunications, Beijing 100876, China; chenyansi@bupt.edu.cn (S.C.); baoyuqi@bupt.edu.cn (Y.B.); xingyaowen@bupt.edu.cn (Y.X.)

**Keywords:** SM4, white-box cryptography, differential computation attack, nonlinear encoding, algebraic attack resistance

## Abstract

Differential Computation Analysis (DCA) leverages memory traces to extract secret keys, bypassing countermeasures employed in white-box designs, such as encodings. Although researchers have made great efforts to enhance security against DCA, most solutions considerably decrease algorithmic efficiency. In our approach, the Feistel cipher SM4 is implemented by a series of table-lookup operations, and the input and output of each table are protected by affine transformations and nonlinear encodings generated randomly. We employ fourth-order non-linear encoding to reduce the loss of efficiency while utilizing a random sequence to shuffle lookup table access, thereby severing the potential link between memory data and the intermediate values of SM4. Experimental results indicate that the DCA procedure fails to retrieve the correct key. Furthermore, theoretical analysis shows that the techniques employed in our scheme effectively prevent existing algebraic attacks. Finally, our design requires only 1.44 MB of memory, significantly less than that of the known DCA-resistant schemes—Zhang et al.’s scheme (24.3 MB), Yuan et al.’s scheme (34.5 MB) and Zhao et al.’s scheme (7.8 MB). Thus, our SM4 white-box design effectively ensures security while maintaining a low memory cost.

## 1. Introduction

In traditional cryptographic analysis, it is assumed that attackers can only access the input and output of the cryptographic procedure, which is executed in a secure environment, known as the black-box attack model. However, due to the diverse deployment environments of digital products, cryptographic algorithms are often executed in untrusted settings, resulting in the potential for secure information leakage. In 2002, Chow et al. introduced the concept of a white-box attack environment [1], in which an attacker has full access to memory data during the software’s execution. To mitigate the risks posed by white-box attackers, they developed white-box implementations of the Data Encryption Standard (DES) [1] and the Advanced Encryption Standard (AES) [2]. In their white-box implementation of AES, the operations within the round function were implemented using table lookup operations. Invertible linear transformations and nonlinear encodings were employed to obfuscate the input and output of each table, thereby preventing the leakage of intermediate data during the encryption process.

### 1.1. Related Work

(1) The following research has been conducted on AES white-box schemes. In 2004, Billet et al. introduced the BGE attack method [3], successfully extracting the key from the AES white-box scheme proposed by Chow et al. The BGE method is an algebraic analysis, necessitating the attacker to understand the detailed implementation steps through complex reverse engineering.

In 2019, Bos et al. introduced Differential Computation Analysis (DCA) to extract keys from white-box schemes [4]. DCA utilizes tools to capture traces of memory information during software execution, significantly reducing the workload of reverse engineering. Currently, many white-box schemes, including those submitted to the WhibOx 2016 white-box cryptography competition, have been successfully compromised using DCA. Consequently, DCA poses a significant challenge to white-box implementations.

In response to DCA attacks, several schemes have been proposed. In 2018, Bock et al. evaluated common protection methods in white-box implementations, such as linear transformations and 4-bit nonlinear encodings [5], concluding that neither was effective in resisting DCA. In 2020, Lee et al. improved their AES white-box implementation [6] by using linear Boolean masking to obfuscate the results of all lookup tables [7], successfully thwarting DCA. However, this scheme required significant memory to mask and unmask the genuine internal data. In the same year, Biryukov et al. applied nonlinear masking combined with Boolean masking to obfuscate intermediate values [8], which also effectively prevented DCA. Despite that, this approach significantly increased memory consumption, and its security against algebraic attacks remains unverified.

(2) The following research has been conducted on SM4 white-box schemes. SM4 is a Feistel cipher that was published in 2006 as a Chinese National Standard. In 2021, it was officially published as an ISO/IEC international standard. It has been integrated into the ARMv8.4-A, and support for the RISC-V architecture was ratified in 2021.

In 2009, Xiao et al. introduced the first white-box implementation of SM4 [9], referred to as the Xiao–Lai scheme. This approach utilized external encoding to protect both plaintext and ciphertext, as well as affine transformations to secure intermediate data during the encryption process. Although the scheme is designed to thwart BGE attacks, Lin et al. demonstrated in 2013 that they could successfully extract the key [10] from the Xiao–Lai scheme using a combination of the differential analysis and BGE attacks, with a time complexity of $2^{47}$, referred to as Lin–Lai analysis.

In 2015, Bai et al. introduced the Bai–Wu scheme, which employed complex internal encodings to enhance security [11] against Lin–Lai analysis. However, generating a white-box instance of this scheme required 32.5 MB of memory. In 2018, Pan et al. introduced a new analysis technique [12], denoted as Pan analysis, which demonstrated that the complex internal encodings of the Bai–Wu scheme provided only limited security benefits. Also, in 2015, Shi et al. proposed an SM4 white-box scheme utilizing dual ciphers and random obfuscation to protect lookup tables [13], claiming it could resist Lin–Lai analysis. However, since the author of this scheme did not provide an open-source procedure, no analytical results regarding its security are presently accessible. In 2020, Yao introduced a new SM4 white-box scheme that employed internal state expansion in combination with random numbers to obfuscate the keys [14]. This approach significantly increased the difficulty of key extraction through algebraic analysis methods. So far, no analytical results regarding its security have been published.

In addition to algebraic analysis methods, DCA also poses a significant threat to SM4 white-box designs. In 2022, Zhang et al. [15] introduced intermediate-value mean differential analysis (IVMDA), a technique based on DCA, which successfully extracted the key from the Xiao–Lai scheme. In 2023, Yuan et al. [16] proposed an enhanced DCA technique that successfully compromised the Bai–Wu scheme.

To counter DCAs, several SM4 white-box schemes have been proposed in recent years. In 2022, Zhang et al. introduced an SM4 white-box implementation that enhanced the Xiao–Lai scheme by incorporating 8-bit nonlinear encodings [15], referred to as Zhang’s scheme. Experimental results from IVMDA demonstrated that the scheme could resist DCA. However, the use of 8-bit nonlinear encodings significantly increased the memory consumption to 24.3 MB. In 2023, Yuan et al. [17] proposed improvements to the Bai–Wu scheme, referred to as Yuan’s scheme. They applied protection only to the first and last rounds of the algorithm to reduce memory usage, as DCA primarily targets key-related lookup tables in these iteration rounds. Despite this optimization, this scheme still required 34.5 MB of memory. In 2024, Zhao et al. introduced an SM4 white-box scheme based on the Xiao–Lai approach, utilizing Boolean masking techniques [18], referred to as Zhao’s scheme. This scheme employs nonlinear permutations to reuse random mask values, reducing memory consumption. Two versions were proposed: a simplified version that applies masking to only the first and last four rounds, requiring 1.62 MB of memory, and an enhanced version that applies masking to all rounds, requiring 7.8 MB. The latter was shown to resist both DCA and existing algebraic attacks.

At present, three schemes are capable of resisting both DCA and algebraic analysis, i.e., the schemes proposed by Zhang et al. [15], Yuan et al. [16], and Zhao et al. [18], respectively. However, if the three schemes implement full-round defense, their memory usage tends to be high, resulting in significantly high implementation costs.

### 1.2. Our Contribution

This paper introduces an SM4 white-box scheme that is implemented through a series of table lookup operations. The scheme employs affine transformations and fourth-order nonlinear encodings to protect the input and output of each table. To further enhance security, random sequences are used to shuffle the execution order of the table lookups during the encryption process.

As shown in Table 1, the scheme requires a total of 1.44 MB of memory, which is significantly less than other DCA-resistant methods: one-twelfth of the memory required by Zhang’s scheme [15], one-eighteenth of the memory required by Yuan’s scheme [17], and one-fourth of the memory required by Zhao’s scheme [18]. Furthermore, it takes 44 ms to generate a white-box encryption instance and only 2 ms to encrypt a plaintext block on a personal computer.

Experimental results using the open-source tool Deadpool confirm that the proposed scheme is resistant to DCA. Additionally, theoretical analysis demonstrates that it withstands known algebraic attacks, such as BGE analysis [3], Lin–Lai analysis [10], and Pan analysis [12]. As shown in Table 1, while Zhang’s, Yuan’s, Zhao’s, and our schemes are all secure against both algebraic attacks and DCA, our scheme achieves this with the lowest memory consumption.

### 1.3. Organization

The rest of this paper is organized as follows. The preliminaries are introduced in Section 2. Section 3 explains the basic idea and detailed steps of our SM4 white-box algorithm. Section 4 evaluates the performance of the scheme and compares it with other SM4 white-box algorithms. Algebraic analysis and DCA analysis are conducted in Section 5. Finally, we conclude the paper in Section 6.

## 2. Preliminaries

We modify the Xiao–Lai solution to resist DCA. Here, we briefly introduce the SM4 algorithm and the Xiao–Lai SM4 white-box algorithm.

### 2.1. SM4 Algorithm

SM4 is a Feistel cipher in which the block size and the key length are 128 bits. The encryption process consists of 32 rounds of iterations, and each round requires a 32-bit round key.

The 128-bit plaintext is divided into four 32-bit words $\left(X_{0},X_{1},X_{2},X_{3}\right)$. The round function *F* takes four intermediate state words and the round key and returns a new word. The round function for the $i^{th}$ round iteration is computed as follows:

(1) $$X_{i+4}=F\left(X_{i},X_{i+1},X_{i+2},X_{i+3},rk_{i}\right)=X_{i}⊕T\left(X_{i+1}⊕X_{i+2}⊕X_{i+3}⊕rk_{i}\right).$$

Here, *T* consists of a nonlinear transformation τ and a linear transformation *L*. Let $A=X_{i+1}⊕X_{i+2}⊕X_{i+3}⊕rk_{i}$ represent the input word of τ, and let *B* be the output. τ involves four independent S-box substitutions, i.e.:

(2) $$B=\left(b_{0},b_{1},b_{2},b_{3}\right)=\tau \left(A\right)=\left(S\left(a_{0}\right),S\left(a_{1}\right),S\left(a_{2}\right),S\left(a_{3}\right)\right).$$

Here, each $a_{j}$ or $b_{j}$(j∈{0,1,2,3}) is a byte.

Both the input and output of the linear transformation *L* are 32-bit values, and the transformation is defined as the following formula:

(3) $$X_{i+4}=L\left(B\right)=B⊕\left(B⋘2\right)⊕\left(B⋘10\right)⊕\left(B⋘18\right)⊕\left(B⋘24\right).$$

**Figure 1:**
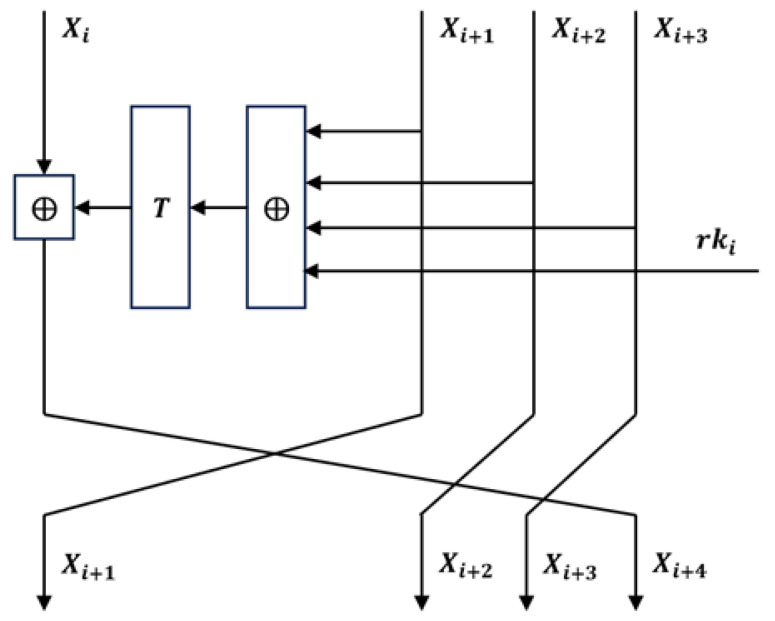
The flowchart of $i^{th}$ round iteration of SM4.

Here, ⋘ represents a cyclic left shift. The flowchart for the *i^th^* round iteration is shown in Figure 1.

After the final round, the result undergoes a simple reverse transformation to produce the final ciphertext $\left(X_{35},X_{34},X_{33},X_{32}\right)$.

### 2.2. Xiao–Lai Scheme

**Figure 2:**
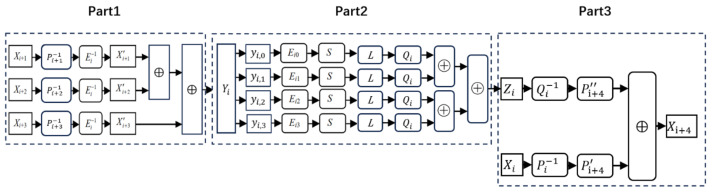
The $i^{th}$ round function of the Xiao–Lai scheme.

In the Xiao–Lai scheme, the standard SM4 round function is divided into three parts. As shown in Figure 2, the first part computes the XOR of three state words. The second part includes the addition of the round key and the *T* transformation. Finally, the third part calculates the sum of the current intermediate state word and the state word $X_{i}$.

Unlike the original SM4, each intermediate state word is protected by a reversible affine transformation *P* defined as follows:

(4) P(x)=lP×x⊕cP.

Here, lP is a 32-dimension invertible matrix over GF(2), and cP is a 32-dimension constant vector over GF(2). Consequently, each part of the process also involves removing the previous affine transformation and applying a new one.

**Part 1:** Computing $Y_{i}=X_{i+1}⊕X_{i+2}⊕X_{i+3}$.

Part 1 consists of three affine transformations and two XOR operations. As mentioned before, the state words are protected by affine transformations, denoted by $X_{i+1}^{\prime },X_{i+2}^{\prime },X_{i+3}^{\prime }$, so the inverse transformation $P_{i+j}^{-1}\left(j=1,2,3\right)$ should be applied first. To avoid the leakage of $P_{i+j}^{-1}\left(j=1,2,3\right)$, the same affine transformation $E_{i}^{-1}$ is separately merged into $P_{i+1}^{-1}$, $P_{i+2}^{-1}$, and $P_{i+3}^{-1}$. Thereby, a compounded transformation $E_{i}^{-1}\circ P_{i+j}^{-1}$ is applied to state word $X_{i+j}(j\in \left\{1,2,3\right\}$, called the encoding unification operation. It is notable that $E_{i}=\mathrm{diag}\left(E_{i0},E_{i1},E_{i2},E_{i3}\right)$, where each $E_{ij}$ is an 8-order reversible affine transformation over GF(2), and $E_{i}^{-1}$ is the inverse transformation of $E_{i}$.

Now, the three words are secured by the same transformation $E_{i}^{-1}$, the XOR addition can be computed directly, and the result of part one is protected by the affine transformation $E_{i}^{-1}$. The overall computation process is as follows:

(5) $$\begin{array}{cc} Y_{i}= & E_{i}^{-1}\left(X_{i+1}⊕X_{i+2}⊕X_{i+3}\right)=\left(E_{i}^{-1}\circ P_{i+1}^{-1}\right)X_{i+1}^{\prime }\\ \; & ⊕\left(E_{i}^{-1}\circ P_{i+2}^{-1}\right)X_{i+2}^{\prime }⊕\left(E_{i}^{-1}\circ P_{i+3}^{-1}\right)X_{i+3}^{\prime }. \end{array}$$

**Part 2:** The round key addition and *T* transformation.

All the operations included in this part are implemented using four table lookups and three XOR operations. Since *T* is a 32-bit-to-32-bit transformation, creating a single lookup table would consume too much memory. Therefore, it is split into four 8-bit-to-32-bit lookup tables. Each table is created according to the following equation:

(6) $$Z_{i,j}=Q_{i}\circ L\circ Sbox\left(\left(E_{i,j}\cdot y_{i,j}\right)⊕{rk}_{i,j}\right).$$

Here, $y_{i,j}$ is the *j^th^* byte of the output of Part 1, and $rk_{i,j}$ is the the *j^th^* byte of the *i^th^* round key. Similarly, decoding the protection $E_{i}^{-1}$ and adding new protection $Q_{i}$ are necessary separately before and after the round operations.

Also, the output values of the four tables are protected by the same affine transformation $Q_{i}$. Thus, the results of the four table lookups are XORed directly to obtain the output $Z_{i}$ of Part 2.

(7) $$Z_{i}=Q_{i}\left(T\left(X_{i+1}⊕X_{i+2}⊕X_{i+3}⊕rk_{i}\right)\right)=Z_{i,0}⊕Z_{i,1}⊕Z_{i,2}⊕Z_{i,3}.$$

**Part 3:** Adding $X_{i+4}$.

This part consists of two affine transformations and one XOR operation. $X_{i}^{\prime }$ and $Z_{i}$ are protected by different affine transformations, so the encoding unification operation should be executed before the XOR operation. However, if two values are protected by the same affine transformation, the XOR sum will only be protected by the linear component of the affine transformation. Therefore, two affine transformations $P_{i+4}^{\prime }\left(x\right)=lP_{i+4}\times x⊕cP_{i+4}^{\prime }$ and $P_{i+4}^{\prime \prime }\left(x\right)=lP_{i+4}\times x⊕cP_{i+4}^{\prime \prime }$ are chosen. The linear components of $P_{i+4}^{\prime }$ and $P_{i+4}^{\prime \prime }$ are the same as those of $P_{i+4}$, but the constant components differ and satisfy $cP_{i+4}^{\prime }+cP_{i+4}^{\prime \prime }=cP_{i+4}$.

Consequently, transformations $P_{i+4}^{\prime }\circ P_{i}^{-1}$ and $P_{i+4}^{\prime \prime }\circ Q_{i}^{-1}$ are separately applied to $X_{i}$ and $Z_{i}$. The output of Part 3 is protected by $P_{i+4}$, i.e.,

(8) $$X_{i+4}^{\prime }=P_{i+4}\left(X_{i}⊕T\left(X_{i+1}⊕X_{i+2}⊕X_{i+3}⊕rk_{i}\right)\right).$$

## 3. Improved SM4 White-Box Scheme

**Figure 3:**
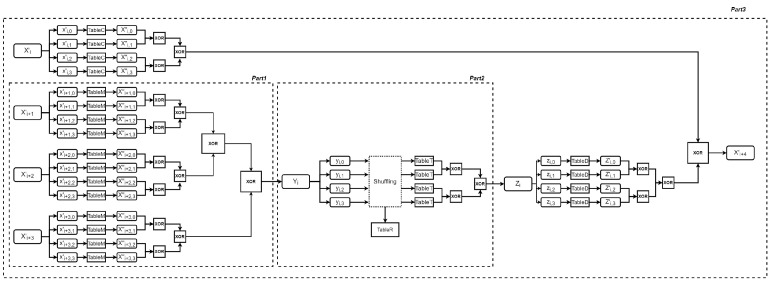
Round function of our SM4 white-box scheme.

Our proposal builds upon the design of the Xiao–Lai scheme, with the round function similarly divided into three parts, as illustrated in Figure 3. We begin by defining the notations used throughout the paper (see Table 2), followed by a brief introduction to our design concept. Lastly, we provide a detailed explanation of the construction of each part.

### 3.1. Design Ideas

According to the algebraic analyses presented separately by Pan et al. [12] and Lin et al. [10], the combination of the last two parts and Part 1 of the next round of iteration in the Xiao–Lai scheme may expose intermediate affine transformations, allowing an attacker to recover the key. Hence, we add nonlinear encodings to the input and output of intermediate state words to reduce the potential correlations between the genuine and observed values of state words, thus mitigating the previous algebraic attacks. In this context, the nonlinear encoding is a randomly generated table representing a permutation of the set {0,1,…,15}.

However, nonlinear encoding makes directly computing the XOR sum of two state words infeasible, although the affine transformations protecting the two words are unified. Thereby, an XOR table is utilized to achieve XOR operations between the two words. If an 8-to-8-bit nonlinear encoding is used, the XOR table consumes $2^{8}\times 2^{8}\times 8$ bits = $2^{6}$ KB of memory, while a 4-to-4-bit nonlinear encoding requires only $2^{4}\times 2^{4}\times 4$ bits = 0.125 KB. As a result, we adopt eight independent 4-to-4-bit nonlinear encodings to secure each intermediate state word throughout the encryption process, minimizing memory consumption.

Furthermore, the success of DCA relies on aligned memory traces, so it would be great if we could intentionally perturb the trace alignment. Meanwhile, the four lookup tables within Part 2 of the Xiao–Lai scheme are crucial because the round key is hidden in the table. Especially, the calculation order of the four lookups can be adjusted. Hence, this scheme introduces a random sequence to shuffle the access order of those tables, dynamically varying data flow processing orders during multiple encryptions.

### 3.2. Construction of Our Scheme

**Part 1:** Computing $Y_{i}=X_{i+1}⊕X_{i+2}⊕X_{i+3}$.

As previously noted, each state word is protected by an affine transformation and eight nonlinear encodings, so the XOR operation is executed through table lookups. However, adding two 32-bit words together would require a table occupying $2^{69}\left(=2^{32}\times 2^{32}\times 32\right)$ bits of memory, which is impractical due to excessive memory demands. To address this, as illustrated in Figure 3, we divide the word-level computation into four independent byte-level computations during the encoding unification process. Then, the addition of two nibbles is computed using an XOR table.

(1) Encoding unification.

In the Xiao–Lai scheme, encoding unification operation applies an affine transformation $E_{i}^{-1}\circ P_{i+j}^{-1}$ to the input $X_{i+j}^{\prime }$. Also, because of non-linear encodings, the affine transformation combined with the inverse of the non-linear encodings is transferred to table lookup operations. To save memory, each 32-bit input $X_{i+j}^{\prime }$ (where j∈{1,2,3}) is split into four concatenated bytes $\left(x_{i+j,0}^{\prime },x_{i+j,1}^{\prime },x_{i+j,2}^{\prime },x_{i+j,3}^{\prime }\right)$. Each byte is processed through an 8-to-32-bit table lookup operation, and then, the resulting four words are added together as follows:

(9) $$X_{i+j}^{\prime \prime \prime \prime }=\overset{3}{\underset{k=0}{⨁}}\left(\left(\begin{array}{c} out_{i+j,k,2k}^{4}\\ out_{i+j,k,2k+1}^{4} \end{array}\right)\circ \left(E{\left[k\right]}_{i}^{-1}\circ P{\left[k\right]}_{i+j}^{-1}\right)\circ \left(\begin{array}{c} in_{i+j,k,0}^{0}\\ in_{i+j,k,1}^{0} \end{array}\right)\circ x_{i+j}^{\prime }\right)$$

**Figure 4:**
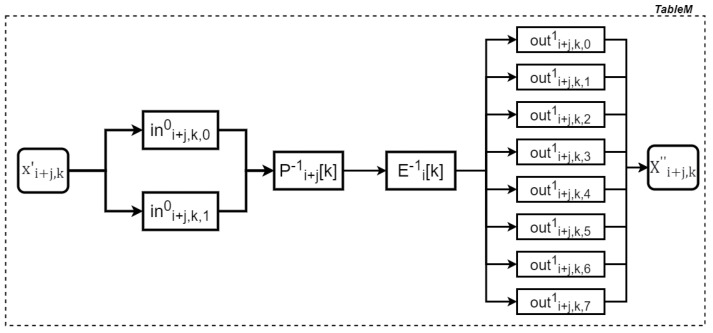
Generating a TableM table.

Here, $P_{i+j}\left[k\right]\left(\cdot \right)$(k∈{0,1,2,3}) refers to the partial computation of affine transformation $(P_{i+j}$). Specifically, the $k^{th}$ eight columns of $lP(P_{i+j}$) are multiplied by the byte vector $x_{i+j,k}$, followed by the addition of the $k^{th}$ eight rows of $cP(P_{i+j}$). The partial computation of affine transformation $E_{i}^{-1}\circ P_{i+j}^{-1}$, along with the associated nonlinear encodings and decodings, is consolidated into a table, referred to as TableM. The process for creating this table is shown in Figure 4.

**Figure 5:**
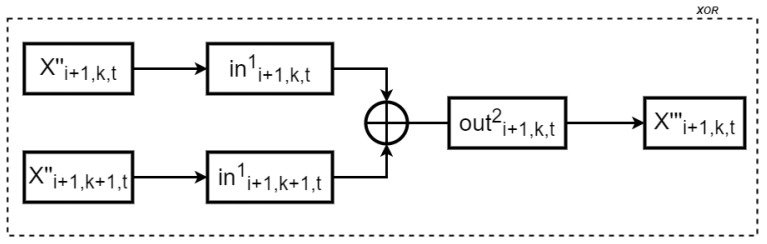
Generating an XOR Table.

The following XOR operations are performed using nine XOR tables, which take two 4-bit inputs and produce a 4-bit output, as illustrated in Figure 5. After removing the nonlinear encoding, the two nibbles are protected by the same affine transformation, ensuring that the XOR sum remains protected by this transformation. Finally, a new nonlinear encoding $out_{i+1,k,t}^{1}$ is applied. Let $X_{i+1,k+1,t}^{\prime \prime }$ represent the $t^{th}$ nibble of word $X_{i+1,k+1}^{\prime \prime }$. Taking $X_{i+1,k+1,t}^{\prime \prime }⊕X_{i+1,k+1,t}^{\prime \prime }$ as an example, the process for generating the XOR table includes the following operations:

(10) $$\left(out_{i+1,k,t}^{2}\right)\left(\left(in_{i+1,k,t}^{1}\left(X_{i+1,k,t}^{\prime \prime }\right)\right)⊕\left(in_{i+1,k+1,t}^{1}\left(X_{i+1,k+1,t}^{\prime \prime }\right)\right)\right).$$

(2) XOR operation.

The XOR operation of Part 1 after the encoding unification also uses lookup tables. Taking $X_{i+1}^{\prime \prime \prime }$ and $X_{i+2}^{\prime \prime \prime }$ as an example, let $X_{i+1,,t}^{\prime \prime \prime }$ and $X_{i+2,,t}^{\prime \prime \prime }$ represent the two $t^{th}$ 4-bit inputs to the XOR table. The table is created according to the following equation.

(11) $$X_{i+1,,t}^{\prime \prime \prime }=out_{i+1,t}^{2}\left(in_{i+1,,t}^{1}\left(X_{i+1,,t}^{\prime \prime \prime }\right)⊕in_{i+1,k+1,t}^{1}\left(X_{i+2,,t}^{\prime \prime \prime }\right)\right)$$

(3) Space complexity.

Part 1 involves two types of lookup tables: the TableM table and the XOR table. Each $X_{i+j}$ (j=1,2,3) can be represented as four concatenated bytes, with each byte serving as the input of a TableM table. Therefore, each round requires 3×4=12 TableM tables, and for 32 rounds of iterations, a total of 12×32=384 tables are needed. A TableM table takes an 8-bit input and returns a 32-bit value. Thus, each TableM table occupies $1\;\mathrm{KB}(=2^{8}\times 32\;\mathrm{bits})$ of memory, so all TableM tables require 384 KB of memory in a white-box instance.

Each 32-bit word $X_{i+j,k}^{\prime \prime }$ is split into eight concatenated 4-bit segments $X_{i+j,k,t}^{\prime \prime }\left(t=0,1,,\ldots ,7\right)$. Two corresponding 4-bit segments from two words are the input to one XOR table. Thus, the addition of two 32-bit words requires eight XOR tables. There are a total of twelve words that require eleven XOR operations. Therefore, Part 1 of each round requires 11×8=88 XOR tables, and for 32 rounds of iterations, a total of 88×32=2816 XOR tables are needed. Each XOR table occupies $2^{4}\times 2^{4}\times 4\;\mathrm{bits}=128\;B$, so in a white-box instance, the XOR tables in Part 1 occupy 32×11×8×128B=352KB of memory.

**Part 2:** The round key addition and *T* transformation.

The T transformation $T(X_{i+1}⊕X_{i+2}⊕X_{i+3}⊕{\mathrm{rk}}_{i})$ is implemented by four table lookups followed by XOR operations in the Xiao–Lai scheme. The round key is embedded during the process of generating the lookup tables. We inherit the method of implementation of the T transformation but add non-linear encodings to further protect intermediate data. Moreover, the order of access to the four tables is randomly shuffled. Also, because of the non-linear encodings, the addition of the four output words from four table lookups is conducted using XOR tables.

(1) Tables embedded with the round key.

**Figure 6:**
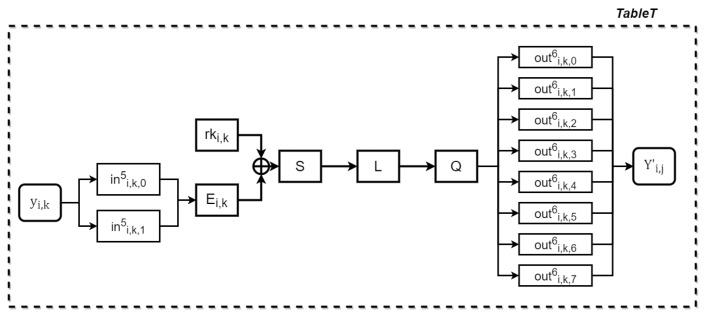
Generating a TableT table.

The output $Y_{i}$ from Part 1 is divided into four 8-bit segments: $y_{i,0}$, $y_{i,1}$, $y_{i,2}$, and $y_{i,3}$. Each byte is then used as input for a lookup table, referred to as a TableT table. As shown in Figure 6, in addition to the operations required to create a table in the Xiao–Lai scheme, nonlinear decoding ${\left(in_{i,k,0}^{5},in_{i,k,1}^{5}\right)}^{T}$ and nonlinear encoding ${\left(out_{i,k,0}^{6},\cdots ,out_{i,k,7}^{6}\right)}^{T}$ are separately applied before and after these operations. The process for generating a TableT table involves the following operations:

(12) $$\begin{array}{cc} Y_{i,k}^{\prime }= & ({\left(out_{i,k,0}^{6},\cdots ,out_{i,k,7}^{6}\right)}^{T}\circ Q_{i}(L\circ Sbox(\\ \; & \left(E_{i,k}\circ {\left(in_{i,k,0}^{5},in_{i,k,1}^{5}\right)}^{T}\left(y_{i,k}\right)\right)⊕{rk}_{i,k})). \end{array}$$

(2) Shuffling.

To prevent DCAs, the query order of the four TableT lookups are randomized using a randomly generated sequence. Typically, the computation order follows $y_{i,0},y_{i,1},y_{i,2},y_{i,3}$. However, after shuffling based on the random sequence $j_{0},j_{1},j_{2},j_{3}$, the access order is updated to $y_{i,j_{0}},y_{i,j_{1}},y_{i,j_{2}},y_{i,j_{3}}$, and the access order of the corresponding TableT tables is adjusted accordingly.

When generating a white-box encryption instance, we use a TableR table that stores all 24 permutations of {0,1,2,3} (since 4!=24). During encryption, a random number temp is generated, and temp(mod24) is used as an index to select a permutation from the TableR table. The bytes of $Y_{i}$ are then reordered based on the selected permutation, and the four TableT tables are queried in the updated order.

For instance, in the $i^{th}$ round, if the permutation {0,3,2,1} is selected by random number temp(mod24), the byte order of $Y_{i}$ is rearranged to $y_{i,0},y_{i,3},y_{i,2},y_{i,1}$, and the TableT tables are queried in that new order—first, fourth, third, and second.

(3) XOR operations.

Each TableT lookup returns a 32-bit word. By performing lookups on four tables, four words are obtained. These words are then XORed using XOR tables to produce the output $Z_{i}$ for Part 2.

(4) Space complexity.

Part 2 involves three types of lookup tables, TableT tables, XOR tables, and a TableR table for permutations. The input of Part 2 is split into four bytes and each byte $y_{i,k}$ corresponds to a TableT table. Therefore, each round requires 4 TableT tables, and for 32 rounds, a total of 4×32=128 tables are needed. Each TableT table takes an 8-bit input and returns a 32-bit output, so it occupies $2^{8}\times 32\;\mathrm{bits}=1\;\mathrm{KB}$. In total, in a white-box instance, the TableT tables occupy 128×1KB=128KB of memory.

The four TableT lookups generate four 32-bit words, which require three XOR operations. Thus, Part 2 of each round needs 3×8=24 XOR tables. For 32 rounds of iterations, 24×32=768 XOR tables are needed. Each XOR table occupies $2^{4}\times 2^{4}\times 4\;\mathrm{bits}=128\;B$ of memory, so in a white-box instance, the XOR tables in Part 2 require 768×128=96KB of memory.

The TableR table stores all 24 permutations of the set {0,1,2,3}. Each permutation requires 1 byte, resulting in a total storage requirement of 24 bytes for TableR table.

**Part 3:** Adding $X_{i+4}$.

(1) Encoding unification and XOR operation.

**Figure 7:**
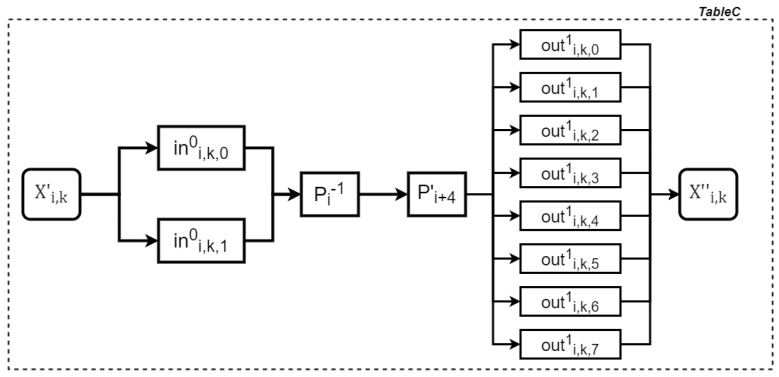
Generating a TableC table.

Part 3 computes the XOR of $X_{i}^{\prime }$ and $Z_{i}$. Similar to Part 1, affine transformation unification is first applied to both words. As a result, four 8-to-32-bit tables are generated for $X_{i}^{\prime }$, called TableC tables, and four for $Z_{i}$, called TableD tables. The process for generating a TableC table involves the steps shown in Figure 7, and the relationship between the input and output of a TableC table is defined as follows:

(13) $$X_{i,k}^{\prime \prime }={\left(out_{i,k,0}^{1},\cdots ,out_{i,k,7}^{1}\right)}^{T}\circ \left(P_{i+4}^{\prime }\circ P_{i}^{-1}\right)\circ {\left(in_{i,k,0}^{0},in_{i,k,1}^{0}\right)}^{T}\left(x_{i,k}^{\prime }\right).$$

**Figure 8:**
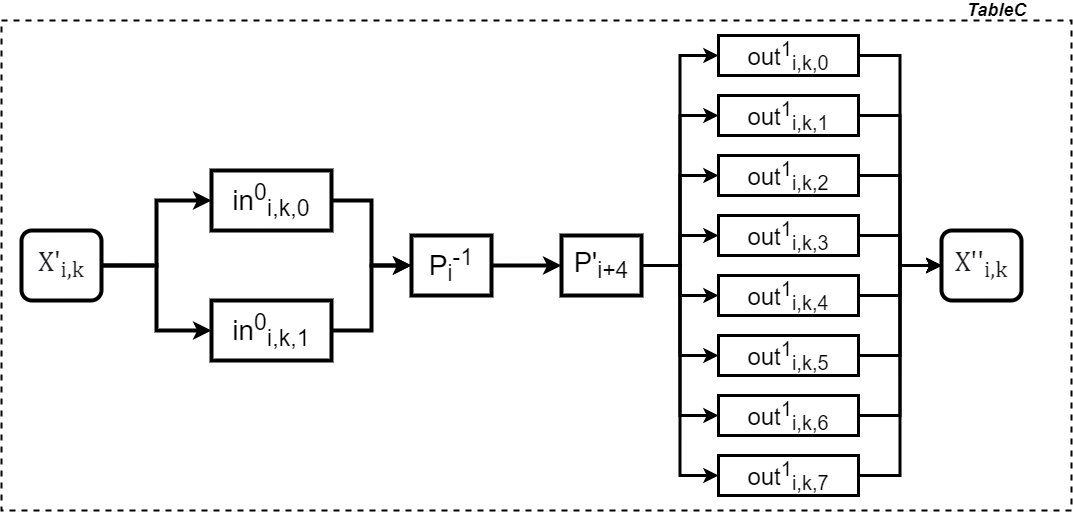
Generating a TableD table.

Similarly, the process of generating a TableD table is demonstrated in Figure 8, and the relationship between the input and output of the table is represented as follows:

(14) $$Z_{i,k}^{\prime }={\left(out_{i,k,0}^{9},\ldots ,out_{i,k,7}^{9}\right)}^{T}\circ \left(P_{i+4}^{\prime \prime }\circ Q_{i}^{-1}\right)\circ {\left(in_{i,k,0}^{8},in_{i,k,1}^{8}\right)}^{T}\left(z_{i,k}\right).$$

As in the Xiao–Lai scheme, the affine transformations $P_{i+4}^{\prime }$ and $P_{i+4}^{\prime \prime }$ share the same linear component, while the sum of their constant components equals the constant component of $P_{i+4}$.

Finally, the eight words $X_{i,k}^{\prime \prime },Z_{i,k}^{\prime }\left(k=0,1,2,3\right)$ are added up with the assistance of XOR tables.

(2) Space complexity.

Part 3 consists of three types of tables: TableC tables, TableD tables, and XOR tables. Both TableC and TableD tables take an 8-bit input and return a 32-bit output. Therefore, each table occupies $2^{8}\times 32\;\mathrm{bits}=1\;\mathrm{KB}$. Each round requires four TableC tables and four TableD tables. Over 32 rounds, this totals 4×32+4×32=256 tables. Consequently, in a white-box instance, the TableC and TableD tables together consume 256×1KB=256KB of memory.

Adding up the eight words $X_{i,k}^{\prime \prime },Y_{i,k}^{\prime }\left(k=0,1,2,3\right)$ requires seven XOR operations, so 8×7=56 XOR tables are used per round. For 32 rounds of iterations, a total of 32×56=1792 XOR tables are needed. The memory required for the XOR tables of Part 3 in a white-box instance is 1792×128B=224KB.

## 4. Performance

The SM4 white-box scheme proposed in this paper utilizes five types of lookup tables: TableM, TableT, TableC, TableD, and XOR tables, along with a permutation table, TableR. The memory usage and quantity of each type of table were calculated in the previous section. As summarized in Table 3, the total memory required to generate an instance of our SM4 white-box scheme is 1.44 MB.

(15) $$\begin{array}{c} 32\;\mathrm{KB}+384\;\mathrm{KB}+128\;\mathrm{KB}+128\;\mathrm{KB}+128\;\mathrm{KB}+672\;\mathrm{KB}=1472\;\mathrm{KB}=1.44\;\mathrm{MB}. \end{array}$$

The round functions in our scheme are executed through a series of table lookup operations, resulting in high-speed encryption performance. We tested the runtime of our proposed white-box scheme on a personal computer with a 12th-generation AMD Ryzen 7 5700U processor (1.80 GHz) with Radeon Graphics and 24 GB of RAM and compared it to other publicly available white-box schemes. The results, presented in Table 4, show that the average runtime to generate a white-box instance of our scheme is approximately 44 ms while encrypting a single block of plaintext takes about 2 ms.

Compared to other SM4 white-box schemes resistant to DCA attacks, our scheme consumes significantly less memory. The schemes proposed by Yuan et al. [17] and Zhang et al. [15] use 8th-order nonlinear encodings, which results in a large size of XOR tables. Similarly, the scheme by Zhao et al. [18] employs a masking technique that necessitates memory for storing lots of randomly generated masking values. As a result, their memory consumption is 23.96 times, 16.86 times, and 5.42 times, respectively, higher than our scheme.

## 5. Security Analysis: White-Box Obfuscation

### 5.1. Algebraic Attacks

#### 5.1.1. BGE Attack

The BGE analysis first combines the lookup tables in the white-box scheme [3]. Therefore, the encodings protected the output of the previous table and the input of the current table are the inverse of each other, and the compound of them are the identity function. The input and output encodings of the non-linear transformation are converted into affine transformations, and then algebraic methods are used to solve the affine transformation. Since the output encoding of each round is the inverse of the input encoding of the next round, the input encoding of all rounds except the first can be obtained by calculating the inverse of the previous round’s output encoding. Therefore, the attacker can solve the hidden key after deducing the encodings protecting the input and output of the combined table and already knowing the round function of the algorithm.

**Figure 9:**
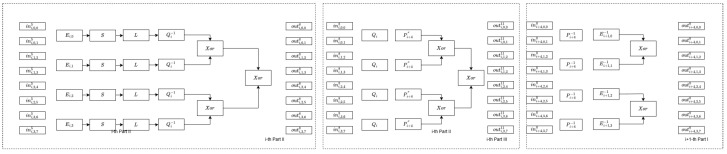
BGE analysis in our scheme.

Similar to the BGE analysis of the Xiao–Lai scheme, the lookup table TableT and XOR tables from Part 2, the lookup table TableC and XOR tables from Part 3, and the lookup table TableM from Part 1 of the subsequent round iteration are combined, as illustrated in Figure 9. In an ideal case, the affine transformation and non-linear encoding protecting the output of one part, such as $Q_{i}^{-1}$ and ${\left(out_{i,k,2k}^{8},out_{i,k,2k+1}^{8}\right)}^{T}$, are the inverses of the non-linear encoding and affine transformation safeguarding the input of the next part, such as $Q_{i}$ and ${\left(in_{i,k,0}^{8},in_{i,k,1}^{8}\right)}^{T}$. Consequently, only known operations like the Sbox and *L* functions remain within the combined table. The attacker then attempts to convert the non-linear encodings protecting the table’s input and output, specifically ${\left(in_{i,k,0}^{0},in_{i,k,1}^{0}\right)}^{T}$ and ${\left(out_{i,k,2k}^{11},out_{i,k,2k+1}^{11}\right)}^{T},k\in \left\{0,1,2,3\right\}$, into affine transformations and solve them.

In the current white-box scheme, the affine transformation and nonlinear encoding used to protect the outputs of Part 3 are not inverse operations for securing the inputs of Part 1 in the next round of iteration. Specifically,

(16) (Pi+4−1[k])ini,k,00ini,k,10∘outi,k,2k11outi,k,2k+111∘(Pi+4′′[k]),k=0,1,2,3;

This means the nonlinear encodings do not cancel each other out, and the combined operation $\left(P_{i+4}^{\prime \prime }\left[k\right]\circ {\left(out_{i,k,2k}^{11},out_{i,k,2k+1}^{11}\right)}^{T}\right)\circ \left({\left(in_{i,k,0}^{0},in_{i,k,0}^{0}\right)}^{T}\circ P_{i+4}^{-1}\left[k\right]\right),k\in \left\{0,1,2,3\right\}$ remains unknown to the attacker. As a result, the BGE attack is effectively thwarted.

#### 5.1.2. Lin–Lai Analysis

The Lin–Lai analysis improves upon the BGE attack by using differential analysis to eliminate the unknown constant. In Lin–Lai analysis against the Xiao–Lai scheme, the operations from Part 2, the encoding unification for $Z_{i}$ from Part 3, and the encoding unification for $X_{i+4}$ from Part 1 of the subsequent round iteration are combined. As shown in Figure 2, $Q_{i}$ and $Q_{i}^{-1}$ can be canceled out, while $P\prime \prime_{i+4}$ and $P_{i+4}^{-1}$ are compounded, leaving only the unknown constant $A_{i+4}$ after the combination. Thus, $X_{i+4}^{\prime }=E_{i+1}^{-1}\left({⨁}_{k=0}^{3}\left[L\circ S\circ E_{i,k}\left(y_{i,k}\right)\right]⊕A_{i+4}\right)$. Furthermore, $E_{i+1}$ are decomposed into operations on individual bytes. The equation can be split into four separate equations as follows:

(17) $$\begin{array}{c} X_{i+4,0}^{\prime }=lE_{i+1,0}^{-1}\left(\overset{3}{\underset{k=0}{⨁}}\left[L\circ S\circ E_{i,k}\left(y_{i,k}\right)\right]⊕g_{i+4,0}\right),\\ X_{i+4,1}^{\prime }=lE_{i+1,1}^{-1}\left(\overset{3}{\underset{k=0}{⨁}}\left[L\circ S\circ E_{i,k}\left(y_{i,k}\right)\right]⊕g_{i+4,1}\right),\\ X_{i+4,2}^{\prime }=lE_{i+1,2}^{-1}\left(\overset{3}{\underset{k=0}{⨁}}\left[L\circ S\circ E_{i,k}\left(y_{i,k}\right)\right]⊕g_{i+4,2}\right),\\ X_{i+4,3}^{\prime }=lE_{i+1,3}^{-1}\left(\overset{3}{\underset{k=0}{⨁}}\left[L\circ S\circ E_{i,k}\left(y_{i,k}\right)\right]⊕g_{i+4,3}\right). \end{array}$$

Here, $g_{i+4,t}\;\left(t\in \left[0,3\right]\right)$ is the sum of $cE_{i+1,t}$ (the constant component of $E_{i+1,t}$) and $A_{i+4,t}$.

Since $X_{i+4,s}^{\prime }$ and $X_{i+4,t}^{\prime }$(s,t∈[0,3]) are affine-related, the affine transformation can first be determined, enabling the recovery of linear components of $E_{i+1,s}^{-1}$ and $E_{i+1,t}^{-1}$, i.e., $lE_{i+1,s}^{-1}$ and $lE_{i+1,t}^{-1}$. Similarly, the linear component of $E_{i,k}$ can be recovered, followed by the linear component of $Q_{i}$. Finally, differential analysis is used to determine the constant components of $E_{i,k}$ and $Q_{i}$, ultimately recovering the key byte.

In our scheme, however, nonlinear encoding is introduced for protection. As previously mentioned, the unknown $\left(P_{i+4}^{\prime \prime }\left[k\right]\circ {\left(out_{i,k,2k}^{11},out_{i,k,2k+1}^{11}\right)}^{T}\right)\circ \left({\left(in_{i,k,0}^{0},in_{i,k,0}^{0}\right)}^{T}\circ P_{i+4}^{-1}\left[k\right]\right)$ after the combination is not equal to a constant but involves nonlinear and affine computations. This prevents the decomposition of the mapping from $y_{i,k}$ to $X_{i+4}^{\prime }$ into the four affine-related equations as per Equation (17). As a result, our scheme is resistant to Lin–Lai analysis.

#### 5.1.3. Pan et al.’s Analysis

Pan et al.’s [12] analysis reduces the complexity of the Lin–Lai analysis by rearranging the recovery order of unknowns. In the Xiao–Lai scheme, Lin et al. [10] first recover the linear component $lE_{i,k}$ and then determine the constant $cE_{i,k}$ using differential analysis, ultimately forming key-related equations to recover the key. Pan et al. [12], however, begin by recovering the constant of each affine transformation and then deduce the linear components using known information.

Whatever analysis method is applied, the three-part combination should first result in four affine-related maps such as Equation (17). As with Lin–Lai analysis, since we added the protection of nonlinear encoding, the compound operations cannot cancel each other out to a constant. Finally, the mapping from $y_{i,k}$ to $X_{i+4}^{\prime }$ cannot be reduced to four affine-related equations. Consequently, Pan et al.’s [12] analysis is thwarted.

### 5.2. DCA Experiments

**Figure 10:**
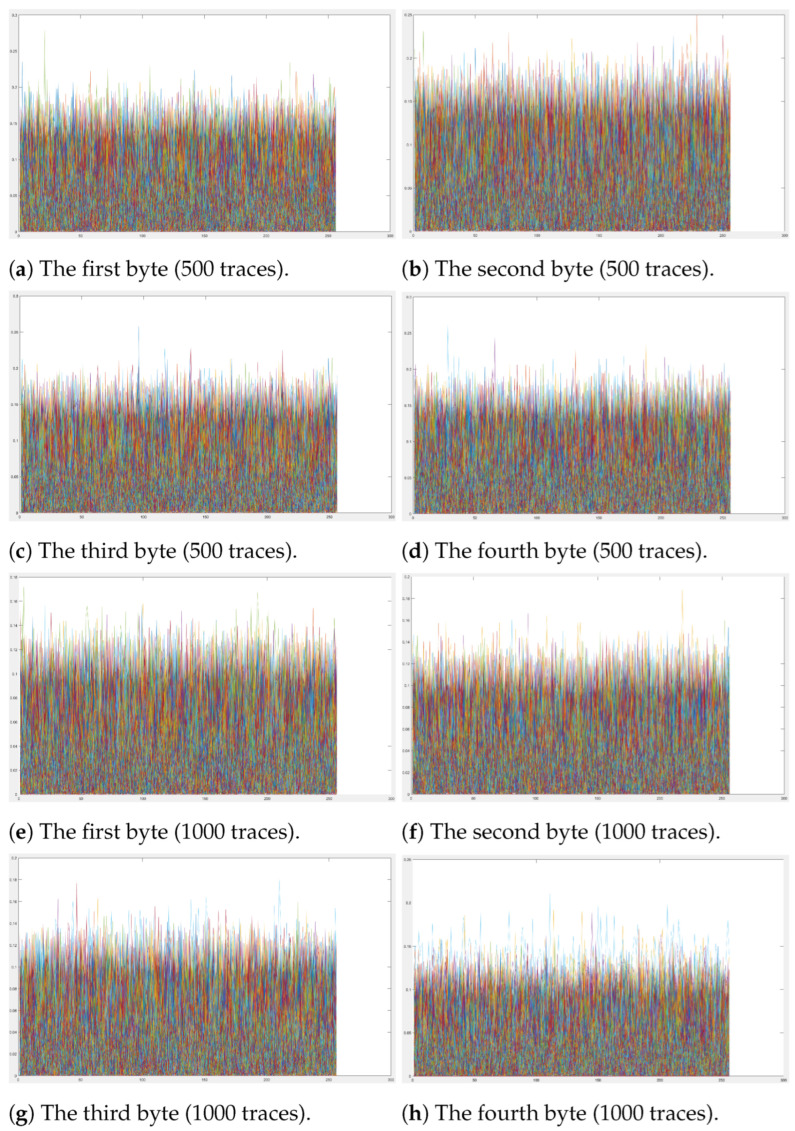
The differential traces related to the first roundkey.

Our proposed scheme employs a shuffling strategy to randomize the execution order of the lookup tables. To evaluate the security of our scheme against DCA, we performed a DCA analysis using the publicly available tool Deadpool [19]. We separately selected 500 and 1000 traces for our experiments. The experimental results, shown in Figure 10, indicate that no significant peaks were observed in the differential traces when analyzing all possible values for each byte of the first-round key. Furthermore, Deadpool failed to return the correct key byte value. These results confirm the security of our scheme against DCA attacks.

## 6. Conclusions

This paper presents an improved SM4 white-box algorithm that addresses the high memory requirements necessary to resist various security threats. The proposed scheme integrates affine and nonlinear encodings to safeguard intermediate data, while a shuffling strategy is employed to prevent the alignment of memory traces during the encryptions of blocks. We evaluated the security of the scheme through existing algebraic attack methods and conducted DCA experiments. The results confirm that the scheme is secure against both algebraic attacks and DCA. Notably, our scheme requires only 1.44 MB of memory, significantly less than other DCA-resistant schemes.

Given ongoing advancements in side-channel analysis techniques, improving the classical DCA approach poses an interesting problem for future research. Additionally, further optimizing the SM4 white-box algorithm for stronger security and greater efficiency remains an open challenge.

## Figures and Tables

**Table 1:** Comparison of white-box implementations.

Scheme	BGE Analysis	Lin–Lai Analysis	Pan Analysis	DCA	Memory
Xiao–Lai scheme [9]	Yes	No	No	No	148.625 KB
Bai–Wu scheme [11]	Yes	Yes	No	No	32.5 MB
Yuan’s scheme [17]	Yes	Yes	Yes	Yes	34.5 MB
Zhang’s scheme [15]	Yes	Yes	Yes	Yes	24.3 MB
Zhao’s scheme [18]	Yes	Yes	Yes	Yes	7.8 MB
Our Scheme	Yes	Yes	Yes	Yes	1.44 MB

“Yes” indicates the scheme can resist this kind of attack; “No” means it cannot.

**Table 2:** Symbols.

Symbol	Description
*i*	The index of the current round of iteration, i=0,…,31.
*j*	The index of a 32-bit word within a 128-bit state input to the round function, j=0,…,3.
*k*	The index of a byte within a state word, k=0,…,3.
*t*	The index of a nibble within a state word, t=0,…,7.
$X_{i+j}$	The $j^{th}$ word input into the $i^{th}$ round of iteration.
$X_{i+j}^{\prime }$	$X_{i+j}$ protected by encodings.
$x_{i+j,k}^{\prime }$	The $k^{th}$ byte of the word $X_{i+j}^{\prime }$.
$rk_{i,k}$	The $k^{th}$ byte of the $i^{th}$ round key.
$X_{i+4}$	The output word of the $i^{th}$ round of iteration.
$Y_{i}$	The output word of Part 1 during the $i^{th}$ round of iteration.
$y_{i,k}$	The $k^{th}$ byte of the word $Y_{i}$.
$Z_{i}$	The output word of Part 2 during the $i^{th}$ round of iteration.
$z_{i,k}$	The $k^{th}$ byte of the word $Z_{i}$.
$P_{i+i}$	A 32-dimensional invertible affine transformation to protect word $X_{i+j}$.
$lP_{i+j}$	The linear component of the affine transformation $P_{i+j}$.
$cP_{i+j}$	The constant component of the affine transformation $P_{i+j}$.
$P_{i+j}^{-1}$	The inverse of the affine transformation $P_{i+j}$.
$E_{i}$	A 32-dimensional affine transformation generated by $diag(E_{i,0},E_{i,1},E_{i,2},E_{i,3})$.
$E_{i,k}$	An 8-dimensional reversible affine transformation.
$E_{i}^{-1}\circ P_{i+k}^{-1}$	The compound affine transformation combining $E_{i}^{-1}$ and $P_{i+k}^{-1}$.
$Q_{i}$	A 32-dimensional invertible affine transformation.
*m*	The index of non-linear encoding, m=0,…,11.
${\mathrm{out}}_{i,k,t}^{m}$	The $t^{th}$ 4-order nonlinear encoding to protect the output word of the current table lookup operation.
${\mathrm{in}}_{i,k,t}^{m}$	The $t^{th}$ 4-order nonlinear decoding to offset the protection of the previous table lookup operation.

**Table 3:** Memory required for tables in our scheme.

Table	Memory	Number of	Memory
	**(Single)**	**Tables**	**(Total)**
TableSE	1 KB	16×1	16 KB
TableFE	1 KB	16×1	16 KB
TableM	1 KB	4×3×32	384 KB
TableT	1 KB	4×32	128 KB
TableC	1 KB	4×32	128 KB
TableD	1 KB	4×32	128 KB
XOR	0.125 KB	32×(8×11+8×3+8×7)	672 KB
TableR	0.375 KB	1	0.375 KB
Total	N/A	N/A	1472 KB

**Table 4:** Performance comparison of various SM4 white-box schemes.

Scheme	Memory	Generation Time	Total Tables	Total XORs	Affine Transformation	Encryption Time
	**(One WB Instance)**	**(s)**	**(8-to-32-bit)**	**(32-bit)**		**(ms)**
Xiao–Lai Scheme [9]	148.625 KB	0.021	128	192	160	0.06 [18]
Bai–Wu Scheme [11]	32.5 MB	3.97	640	640	0	0.001 [18]
Yao’s Scheme [14]	276.625 KB	0.092	128	96 + 96 (64-bit)	160	0.06 [18]
Zhang’s Scheme [15]	24.3 MB	—	640	192	128	—
Yuan’s Scheme [17]	34.5 MB	—	672	536	0	—
Zhao’s Scheme [18]	7.8 MB	2.66	192	208	216	0.08 [18]
Our Scheme	1.44 MB	0.044	800	672	0	2

## Data Availability

The data presented in this study are available on request to the corresponding authors.
